# Low-income community knowledge, attitudes and perceptions regarding antibiotics and antibiotic resistance in Jelutong District, Penang, Malaysia: a qualitative study

**DOI:** 10.1186/s12889-019-7718-9

**Published:** 2019-10-15

**Authors:** Lyna Irawati, Alian A. Alrasheedy, Mohamed Azmi Hassali, Fahad Saleem

**Affiliations:** 1 Faculty of Pharmaceutical Sciences, UCSI University, Kuala Lumpur, Malaysia; 2 School of Pharmaceutical Sciences, Universiti Sains Malaysia, Penang, Malaysia; 3 Unaizah College of Pharmacy, Qassim University, Buraydah, Qassim Saudi Arabia; 4 Faculty of Pharmacy and Health Sciences, University of Balochistan, Quetta, Pakistan

**Keywords:** Antibiotic resistance, Antibiotics, Attitudes, Community, Knowledge, Malaysia, Perceptions, Qualitative study

## Abstract

**Background:**

Understanding community perspectives on antibiotics and antibiotic resistance (ABR) is a key component in designing educational interventions to combat ABR at the community level in Malaysia. Therefore, this study aimed to explore community residents’ knowledge, attitudes and perceptions regarding antibiotics and ABR in Jelutong District, Penang, Malaysia. Moreover, it intended to identify areas of focus to be addressed when designing an educational intervention to increase residents’ knowledge and change their attitudes and perceptions.

**Methods:**

A qualitative approach was adopted to gain a deeper understanding of community residents’ knowledge, attitudes and perceptions regarding antibiotics and ABR. A purposive sampling was employed. Twenty-two residents (aged ≥18 years) were interviewed with the aid of a semi-structured interview guide. All interviews were audio recorded, transcribed verbatim and thematically analysed.

**Results:**

The majority of the participants asserted that antibiotics could be effective against viral infections. Moreover, many participants were unaware that antibiotics have adverse effects. Some acquired antibiotics from a community pharmacy without a prescription, took antibiotics given to them by their family or friends, or took leftover antibiotics prescribed for a previous illness. A few indicated that they would request antibiotics from their physician when they had viral infections. More than half of the participants discontinued taking antibiotics when their symptoms improved. The majority stated that ABR occurs when the body becomes used to antibiotics. Most participants were unaware of the causes, consequences and prevention of ABR. In fact, they were not concerned about it. As a result, only a few perceived themselves as having responsibility for preventing this problem.

**Conclusions:**

The community residents had misconceptions about antibiotics and ABR, negative attitudes towards antibiotics and negative perceptions of ABR. The areas of focus that need to be addressed when designing an educational intervention to increase the general public knowledge and change their attitudes and perceptions are the appropriate use of antibiotics and their adverse effects; the importance of adhering to antibiotic therapy; and the definition, causes, consequences and prevention of ABR.

## Background

Antibiotic resistance (ABR) is a global public health problem that threatens the treatment and prevention of bacterial infections and undermines advanced medical procedures such as cancer chemotherapy, organ transplantations and surgeries [1–3]. ABR can occur anywhere, including in the community, particularly where infections are common and can spread rapidly [1, 4]. Misuse and overuse of antibiotics is accelerating the emergence and spread of ABR [2, 5, 6]. Self-medication with antibiotics and dispensing of antibiotics without a prescription is common in low- and middle-income countries (LMICs) [7–10]. Inappropriate prescribing of antibiotics and suboptimum adherence to antibiotic therapy is not only a frequent occurrence in LMICs, but also in high-income countries [11–14]. Such practices could be due to lack of public awareness regarding ABR and inadequate implementation of regulations on antibiotic prescribing and dispensing [15].

The World Health Organization (WHO), concerned with this growing threat, has developed a global action plan for ABR and urges all nations to increase public knowledge of antibiotics and ABR through effective education and communication [3]. To design effective educational interventions, it is essential to understand public knowledge, attitudes and perceptions regarding antibiotics and ABR [16]. Previous qualitative studies have explored public attitudes towards and perceptions of antibiotics and ABR in European countries [17], India [18], New Zealand [19], Sweden [20], Australia [21], Sri Lanka [22], South Africa [23], Albania [24], Indonesia [25] and the United Kingdom (UK) [26–30]. Overall, these studies indicated that the general public and patients had inadequate knowledge and misconceptions about antibiotics and ABR. Moreover, a considerable proportion of the patients had negative attitudes towards antibiotics and negative perceptions of ABR. For example, some studies showed low knowledge of the differences between viral and bacterial infections among the patients [24, 29]. In addition, the study from Australia showed that the patients’ understanding of ABR is poor and the participants had misconceptions about several aspects of ABR [21]. The study from Sweden reported that the participants considered ABR as a slowly emerging health issue rather than a significant current public health issue that is getting worse [20]. However, the level of knowledge of antibiotics and ABR and the issues surrounding the use of antibiotics can vary from one country to another due to the differences in healthcare systems, educational interventions and public awareness [31, 32].

Based on an extensive search of the literature using several electronic databases (ProQuest, PubMed, Scopus, Web of Science and Wiley Online Library), there is a paucity of data regarding community residents’ knowledge, attitudes and perceptions in relation to antibiotics and ABR in Malaysia. Moreover, it is inappropriate to extrapolate the findings from public attitudes and perceptions conducted in other countries to the local situation because of differences in healthcare systems, contexts and cultures [32]. Therefore, the study aimed to explore community residents’ knowledge, attitudes and perceptions regarding antibiotics and ABR in Jelutong District, Penang, Malaysia. In addition, it intended to identify areas of focus to be addressed when designing an educational intervention to increase the general public knowledge and change their attitudes towards and perceptions. The findings of the study will provide guidance to policymakers and other stakeholders for developing educational interventions that effectively enhance community residents’ knowledge and change their attitudes and perceptions in relation to antibiotics and ABR in Malaysia. Furthermore, it will offer a framework for a national action plan to combat ABR at the community level.

## Methods

### Study design

A qualitative phenomenological approach was employed in this study because it allows the researchers greater freedom in exploring participants’ knowledge, attitudes and perceptions regarding antibiotics and ABR [33–36]. In addition, it is effective for gaining insight into individual experiences, attitudes and perceptions [37, 38]. Moreover, it helps in understanding the meaning, common features or essences of an experience or episode [39, 40].

### Study setting

This study was conducted at 1Malaysia Community in Makloom Street, Jelutong between 4 October and 6 December 2016. Jelutong is an urban district located in North-east Penang. The total number of 1Malaysia Community residents (aged ≥18 years) was approximately 1200 in 2016 [41]. The residents came from different age groups, were multi-ethnic and had varying educational and socioeconomic backgrounds. Most lived in flats in low-rise blocks in Makloom Street, while others lived in landed properties along this street.

The study site (i.e. population) was selected based on several factors. First, the School of Pharmaceutical Sciences, Universiti Sains Malaysia (USM) has good rapport with 1Malaysia Community in Makloom Street. This helped the authors gain access to the field. Second, the community residents showed an interest in the research topic and supported the study. Third, the residents are from different age groups and ethnic, educational and socioeconomic backgrounds. This offered the authors the opportunity to gain a heterogeneous sample. Finally, there was no pre-established relationship between the authors and the participants prior to the study commencing. Thus, participants’ responses would not be distorted and any pre-conceptions would be eliminated from the authors’ understanding of the study phenomena.

### Study participants

Participants were included in the study if they were residents of 1Malaysia Community; were 18 years of age and over; were aware of the term ‘antibiotics’; and were able to speak, read and write in English or Malay. Healthcare professionals, those who did not meet the inclusion criteria or those unwilling to participate were excluded from the study.

### Sampling strategy and sample size

Purposive sampling was adopted in the study because it is the primary sampling technique used in qualitative studies [42–44]. In addition, it helps achieve a representative and heterogeneous sample. Thus, a full spectrum of views can be adequately obtained to reflect the sample’s diversity [45, 46]. The selection of participants was based on age, ethnicity, education level and monthly household income.

Before the data collection began, the first author (LI) met the leader of 1Malaysia Community to inform him of the study, seek his endorsement and discuss any concerns. Since the first author did not know any of the community residents prior to the study commencing, she requested the community leader to nominate those who fulfilled all the study criteria and had the potential to provide rich and relevant data [47–49].

The interviewer (LI) met the nominees and provided them with verbal and written information about the study. Those who agreed to participate were asked to contact the interviewer to arrange an interview. The recruitment continued until data saturation was achieved [50–53], as indicated by data replication or redundancy [50]. In this study, the data collected in the last two interviews were found to be repetitive. Therefore, the sample size of the study was 22 participants. None of the selected participants refused to take part or dropped out of the study.

### Study instrument

A semi-structured interview guide (see Additional file 1) was used to explore participants’ knowledge, attitudes and perceptions regarding antibiotics and ABR. The guide was developed based on an extensive literature review [8, 12, 13, 17–23], and was validated by a panel of experts consisting of two senior academics with expertise in pharmacy practice and a senior academic with expertise in infectious diseases. Minor revisions were made according to their feedback. The guide was then piloted among three community residents. Afterwards it was translated into Malay, the national language of Malaysia, by a certified translator and translated back into English by another independent, certified translator to ensure validity and accuracy. The first author compared the back-translated version with the original version to ensure that an equivalence of meaning between the two languages was achieved [54, 55]. Subsequently, the Malay version was piloted among another three community residents. Minor amendments were made as a consequence of the pilot interviews. Data collected from the pilot interviews were not included in the analysis.

### Data collection

Prior to the interviews, written informed consent was sought and the participants were asked to complete a demographic survey concerning their age, gender, ethnicity, marital status, education level, employment status, monthly household income, the last time they had taken antibiotics and the number of antibiotic courses they had taken in the previous year. Afterwards the interviewer, who is experienced in conducting qualitative interviews and is well-versed in English and Malay, conducted the face-to-face, semi-structured interviews with the aid of the interview guide. Based on the participants’ preference, half of the interviews (*n* = 11) were conducted in English and the other half (*n* = 11) were conducted in Malay. As the relationship with the participants had not yet been established, the interviewer began the interviews by introducing herself and asking some introductory questions to build a rapport with them. The interviews focused on participants’ knowledge, attitudes and perceptions regarding antibiotics and ABR. Appropriate probing questions were asked when necessary. To extricate further ideas from the participants, they were given the freedom to express additional opinions on antibiotics and ABR at the end of the interviews. The interviews were held in 1Malaysia Community hall at a time convenient for the participants. Each interview lasted between 30 and 40 min. Field notes were made during and after each interview. None of the interviews were repeated.

All interviews were audio recorded using a digital voice recorder with the participants’ consent. The recordings were then anonymised and transcribed verbatim by a professional transcriber who is familiar with medical terminology and fluent in English and Malay. The interviewer checked all the transcripts against the audio recordings for accuracy and completeness. When necessary, amendments were made, and then a copy of the transcript was returned to the interviewed participant for verification. All participants endorsed the transcripts without making any corrections. The Malay transcripts (*n* = 11) were then translated into English for analysis.

### Data analysis

All English transcripts (*n* = 22) were thematically analysed by the first author (LI) and an independent researcher with expertise in qualitative research. The analysis began with data familiarisation, a process that involved reading the transcripts repeatedly and noting down ideas. Subsequently, the information pertinent to participants’ knowledge, attitudes and perceptions regarding antibiotics and ABR was identified and coded based on deductive and inductive approaches. These codes were then collated into themes and sub-themes. The first author subsequently discussed the coding, themes and sub-themes with the independent researcher to enhance the data reliability. Afterwards they were reviewed by the co-authors (AAA, MAH and FS). Any discrepancies were resolved by discussion until a consensus was reached. The first author was then presented the findings to the study participants and obtained their feedback to ensure that their perspectives were accurately and clearly represented.

## Results

### Participants’ demographic characteristics

Twenty-two participants (P1–P22) were interviewed. Slightly more than half (*n* = 12) were females. The ages of all participants ranged from 18 to 70 years (Median [Q1-Q3] = 48 [32–64]). They came from a diverse range of ethnic, educational and socioeconomic backgrounds. All participants (*n* = 22) reported that they had previously taken antibiotics. The majority (*n* = 13) reported having taken antibiotics within the previous 6 months, while half (*n* = 11) stated that they had only taken one course of antibiotics in the previous year. Table 1 presents the participants’ demographic characteristics.

**Table 1:** Participants’ demographic characteristics

Variables	n
Gender	
Male	10
Female	12
Age groups (years)	
18–27	5
28–37	5
38–47	6
≥ 48	6
Ethnicity	
Malay	8
Chinese	7
Indian	7
Marital status	
Single	8
Married	8
Separated/divorced	3
Widowed	3
Education level	
Primary education	7
Secondary education	8
Tertiary education (college/university)	7
Employment status	
Employed	6
Student	5
Housewife	5
Retired	6
Monthly household income (MYR)*	
≤ 2000	8
2001–4000	7
> 4000	7
Last time antibiotics were taken	
< 3 months	7
3–6 months	6
7–9 months	5
10–12 months	4
Do not remember	0
Number of antibiotic courses taken in the past year	
One	11
Two	4
Three	4
More than three	3
Do not remember	0

n = number, *1 Malaysian ringgit (MYR) = 0.24 US$ as at 1 September 2018.

### Themes

Four major themes were identified: knowledge of antibiotics; attitudes towards antibiotics; knowledge of ABR; and perceptions of taking responsibility for combating ABR (Table 2).

**Table 2:** Themes and sub-themes generated from the thematic analysis

Themes	Sub-themes
1. Knowledge of antibiotics	a. Understanding the term ‘antibiotics’ and antibiotic use
	b. Knowledge of the adverse effects of antibiotics
2. Attitudes towards antibiotics	a. Attitudes towards acquiring antibiotics
	b. Attitudes towards physicians’ decisions to prescribe antibiotics
	c. Attitudes towards adhering to antibiotic therapy
3. Knowledge of ABR	a. Understanding the term ‘ABR’
	b. Knowledge of the causes of ABR
	c. Knowledge of the consequences of ABR
	d. Knowledge of the prevention of ABR
4. Perceptions of taking responsibility for combating ABR	–

### Theme 1: knowledge of antibiotics

To explore participants’ knowledge of antibiotics, the interviewer asked them about their understanding of the term ‘antibiotics’ and antibiotic use. The interviewer also asked participants about the adverse effects of antibiotics.

### Understanding the term ‘antibiotics’ and antibiotic use

The participants had different understandings of the term ‘antibiotics’ and antibiotic use. The majority had a misconception that antibiotics are medicines that kill viruses and can hasten recovery from viral infections:

*‘Antibiotics are medicines that kill viruses. They are medicines used to speed up recovery from colds and coughs.’ (P7)*

*‘Antibiotics are medicines that kill small living things called viruses. They can cure colds and sore throats quickly.’ (P11)*

Some participants understood antibiotics as medicines that reduce fevers:

*‘As far as I’m concerned, antibiotics are medicines that bring down a high temperature.’ (P2)*

*‘All I know is that antibiotics are medicines used to reduce fevers.’ (P22)*

A few participants understood antibiotics as medicines that relieve pain and reduce inflammation:

*‘I’m completely certain that antibiotics are medicines that ease pain and swelling.’ (P3)*

*‘To my knowledge, antibiotics are the same as painkillers. They reduce pain and swelling.’ (P21)*

Two participants stated that antibiotics are medicines that kill bacteria and can be used to treat bacterial infections:

*‘Antibiotics are medicines that kill germs called bacria [bacteria]. They are effective for the treatment of bacria [bacterial] infections such as umonia [pneumonia].’ (P1)*

*‘Antibiotics are medicines that kill bacteria. They can be used to treat skin infections and urine [urinary tract] infections.’ (P15)*

One participant indicated that antibiotics are medicines that prevent any illnesses from becoming severe:

*‘Antibiotics are pills that prevent all kinds of illnesses from getting worse.’ (P9)*

### Knowledge of the adverse effects of antibiotics

Most participants were unaware that antibiotics have adverse effects; none had experienced adverse effects while taking antibiotics:

*‘I really don’t know whether or not antibiotics have side effects. I’ve never experienced any of them.’ (P2)*

*‘I’m not sure if antibiotics can cause side effects. I have never had side effects from the medicine.’ (P7)*

Only a few participants were aware of some adverse effects of antibiotics such as diarrhoea, stomach discomfort and rashes. They stated that they would seek further consultation with their physician if adverse effects occurred:

*‘As far as I’m concerned, antibiotics can cause side effects such as stomach discomfort. If I experience any side effects, I would go back to my doctor.’ (P14)*

*‘Yes, antibiotics have side effects. If I’m not mistaken, they can cause diarrhoea and rashes. If that happens, I would return to my doctor.’ (P17)*

### Theme 2: attitudes towards antibiotics

To explore participants’ attitudes towards antibiotics, the interviewer asked them how they acquired antibiotics. Participants were also asked about their responses to physicians’ decisions to prescribe antibiotics and their adherence to antibiotic therapy.

### Attitudes towards acquiring antibiotics

The majority of participants reported that they acquired antibiotics from a physician and were advised how to take them:

*‘I never request antibiotics from my doctor. He prescribes them and advises me how to take the medicine.’ (P12)*

*‘My doctor prescribes me antibiotics and advises me how to take them.’ (P22)*

Some participants acquired antibiotics from a community pharmacy before consulting a physician, as they wanted to recover quickly from viral infections. They said that they would consult the physician if symptoms persisted:

*‘When I have a cold, I buy antibiotics in a [community] pharmacy without a prescription because they help me get better. If there is no improvement after taking them, I’ll see a doctor.’ (P7)*

*‘When I have a sore throat, I go to a [community] pharmacy and ask for antibiotics as I want to get better quickly. If my condition doesn’t improve after taking them, I’ll see a doctor for advice.’ (P10)*

A few participants took antibiotics given to them by their family or friends, as they believed that antibiotics are effective for treating colds and sore throats:

*‘I take an antibiotic given by my mum. She said the medicine is effective against colds. I try it and it works.’ (P4)*

*‘When my throat is a bit painful, my girlfriend gives me antibiotics. She said they are good for sore throats. I believe what she said and take the medicine according to her advice.’ (P11)*

Two participants indicated that they took leftover antibiotics prescribed for a previous illness when similar symptoms arose, as they wanted to save the time it would take to visit a primary-care clinic:

*‘If I have similar symptoms, I don’t want to see a doctor because of a long wait in the clinic. I would rather take leftover antibiotics prescribed for my past illness.’ (P8)*

*‘When I have the same symptoms, I usually take leftover antibiotics prescribed for my previous illness. I can’t stand the long waiting time in the clinic.’ (P16)*

### Attitudes towards physicians’ decisions to prescribe antibiotics

When asked about their responses to physicians’ decisions to prescribe antibiotics, the majority stated that they would abide by their physician’s decisions and would not request antibiotics, as they trust the physician:

*‘I trust my doctor. If she doesn’t prescribe antibiotics, I won’t ask for such medicines. I’ll take any medicines given by her.’ (P5)*

*‘I have confidence in my doctor. Whether or not he prescribes antibiotics, I’ll abide by his decision. I won’t demand antibiotics.’ (P19)*

Some participants stated that they would not express their expectations openly to their physician nor request antibiotics, as they wanted to establish a good relationship with the physician:

*‘Although I want antibiotics, I don’t dare to ask my doctor to prescribe the medicine. I would rather build a good relationship with the doctor.’ (P2)*

*‘Deep down, I wish to take antibiotics when I’m not feeling well. But, if my doctor doesn’t prescribe the medicine, I won’t request them. I prefer to have a good relationship with the doctor.’ (P9)*

A few participants indicated that they would make their expectations explicit and request antibiotics from their physician when they had viral infections, as they believed the medicine promotes rapid recovery. They also said that they would consult another physician if their request was not granted:

*‘If I have a cold or sore throat, I’ll certainly expect antibiotics from my doctor as the medicine will help me get over it faster. If he refuses to prescribe antibiotics, I’ll see another doctor.’ (P4)*

*‘When I have a cold, I ask my doctor to prescribe antibiotics because I want to get well quickly. If he is reluctant to do so, I’ll consult another doctor.’ (P11)*

### Attitudes towards adhering to antibiotic therapy

When asked about how they took antibiotics, more than half of the participants stated that they adhered to antibiotic regimens. However, they discontinued taking the antibiotics when their symptoms improved, as they were unaware of the importance of taking the full prescribed course. They believed that they had recovered from the infection when they felt better:

*‘I follow the instructions on the label. But, I stop taking the antibiotics when I feel better. Why should I finish the prescribed course when I have recovered from the infection? I don’t understand!’ (P7)*

*‘I take antibiotics according to the instructions on the label. But, I stop taking them when I feel better. Honestly, I don’t see the point why I should take the full prescribed course when symptoms have cleared up.’ (P16)*

Some participants reported that they occasionally missed a few doses of antibiotics because of their hectic lifestyle. Nevertheless, they were aware of what they should do if they missed a dose and completed the prescribed course:

*‘I occasionally miss one or two doses of antibiotics as I am busy with many things, but I always complete the prescribed course. If I miss a dose, I take the missed dose as soon as I remember, and then continue with the regular [dosage] schedule. I never take a double dose to compensate for the missed dose.’ (P2)*

*‘I try my best to follow the instructions on the label. But, sometimes I forget to take one or two doses because I have a very busy life. If I miss a dose and it is almost time for the next dose, I skip the missed dose and then continue with the regular [dosage] schedule until I finish them all. I never take a double dose to make up for the missed dose.’ (P19)*

Only a few participants stated that they adhered to antibiotic regimens and completed the prescribed course to ensure that all bacteria had been killed:

*‘I take antibiotics according to the doctor’s instructions and finish the prescribed course. Hopefully, all bacteria are killed.’ (P15)*

*‘I follow the doctor’s instructions on how to take antibiotics. I finish them all, even if I feel better because I want to make sure all bacteria are killed.’ (P18)*

Those who did not complete the prescribed course were asked what they would do with the leftover antibiotics. Most participants indicated that they kept them in a cupboard and eventually disposed them of in a dustbin; they had no intention of saving them for future use:

*‘I put the leftover antibiotics in a drawer until a later clear out. When I’m free, I throw them away in a dustbin. I won’t take them at a later date.’ (P13)*

*‘I keep the leftover antibiotics in a cabinet, but I won’t take them next time I’m not well. Eventually I’ll throw them out in a rubbish bin.’ (P21)*

Two participants reported that they did keep the leftover antibiotics for future use:

*‘I keep the leftover antibiotics and take them now and then when I have similar symptoms.’ (P8)*

*‘I keep the leftover antibiotics in a cupboard and use them in the future.’ (P16)*

### Theme 3: knowledge of ABR

To explore participants’ knowledge of ABR, the researcher asked them about their understanding of the term ‘ABR’ and its causes, consequences and prevention.

### Understanding the term ‘ABR’

Most participants had heard of the term ‘ABR’, but only two understood it as a condition where bacteria become resistant to antibiotics:

*‘In my opinion, antibiotic resistance occurs when bacteria are able to resist the effects of antibiotics.’ (P1)*

*‘As far as I’m concerned, antibiotic resistance is a condition where bacteria are able to protect themselves from the effects of antibiotics.’ (P5)*

Three participants were unaware of the term:

*‘Antibiotic resistance? What’s that? I don’t have a clue!’ (P4)*

*‘Huh? This is the first I’ve heard of the term [antibiotic] resistance.’ (P10)*

*‘What? I’m not aware of the term [antibiotic] resistance.’ (P22)*

The majority stated that ABR occurs when the body becomes used to antibiotics:

*‘Antibiotic resistance is a condition where the body becomes used to antibiotics.’ (P6)*

*‘My understanding of antibiotic resistance is that it occurs when the body gets used to antibiotics.’ (P8)*

Other participants understood ABR as follows:

- antibiotics losing their potency before killing bacteria:

*‘I think antibiotic resistance occurs when antibiotics lose their potency before killing bacteria.’ (P21)*

- the body becoming incompatible with antibiotics:

*‘The body is not compatible with antibiotics. That’s what antibiotic resistance is.’ (P13)*

- the illness becoming too strong for antibiotics to manage:

*‘As far as I know, antibiotic resistance is a condition where an illness is becoming stronger than antibiotics.’ (P16)*

- a hereditary illness:

*‘Based on my understanding, antibiotic resistance is a hereditary illness. If none of our family members is affected by antibiotic resistance, we won’t suffer from it.’ (P19)*

### Knowledge of the causes of ABR

Although most participants noted an association between antibiotic use and ABR, they expressed uncertainty about the causes of ABR:

*‘I guess there is a link between antibiotic use and antibiotic resistance. But, I’m not sure how antibiotic resistance arises.’ (P3)*

*‘I don’t really know the root of antibiotic resistance. Perhaps there is a connection between antibiotic use and [antibiotic] resistance.’ (P6)*

Some participants stated that inappropriate prescribing of antibiotics, including unnecessary prescription of this medicine, can lead to ABR:

*‘In my opinion, unnecessary antibiotic prescriptions can contribute to antibiotic resistance.’ (P12)*

*‘I guess over-prescription of antibiotics can lead to antibiotic resistance.’ (P14)*

A few participants attributed ABR to the irresponsible use of antibiotics such as not adhering to antibiotic regimens and not completing the prescribed course:

*‘I think antibiotic resistance is likely to occur if we don’t take antibiotics according to the instructions on the label.’ (P15)*

*‘Antibiotic resistance may occur if we don’t finish the prescribed course of antibiotics.’ (P18)*

Two participants indicated that inadequately implementing the regulations on antibiotic availability could contribute to ABR. They reported that some community pharmacists dispensed antibiotics over the counter (OTC), although in Malaysia, regulations prohibit dispensing antibiotics without a prescription:

*‘When I picked up my dad’s medication from a [community] pharmacy, I saw something was not right. The pharmacist sold antibiotics under the counter. You know, such actions can lead to antibiotic resistance.’ (P17)*

*‘I notice some [community] pharmacists sell antibiotics without a prescription, even though the law says otherwise. I think such things can contribute to antibiotic resistance.’ (P20)*

### Knowledge of the consequences of ABR

Most participants were unaware of the consequences of ABR. Moreover, they were not concerned about it:

*‘I don’t know the consequences of antibiotic resistance. Anyway, it doesn’t concern me at all!’ (P10)*

*‘I can’t think of the consequences of [antibiotic] resistance. In fact, I’m not concerned about it.’ (P13)*

Only two participants were aware of some consequences of ABR, including infections being more difficult to treat and the higher cost of treatment:

*‘As far as I know, illnesses are harder to treat because of [antibiotic] resistance.’ (P1)*

*‘I’m sure the cost of treatment will increase because of antibiotic resistance.’ (P5)*

### Knowledge of the prevention of ABR

Most participants were unaware of how ABR can be prevented. They also indicated that they were not at risk of contracting antibiotic-resistant infections and had a limited role in avoiding the emergence and spread of ABR:

*‘I’m just an ordinary person. I’m not sure how antibiotic resistance can be prevented. I guess there is nothing I can do about it. Anyway, I don’t think antibiotic resistance will affect me.’ (P2)*

*‘I don’t know how we can prevent antibiotic resistance. I guess I can do nothing to slow down the spread of the resistance. Somehow, I believe that I’m not at risk of getting an antibiotic-resistant infection.’ (P6)*

Conversely, only a few participants stated that responsible use of antibiotics, such as adhering to antibiotic regimens and completing the prescribed course, can prevent ABR:

*‘I suppose taking antibiotics exactly as your doctor tells you can prevent antibiotic resistance.’ (P15)*

*‘Finishing the prescribed course of antibiotics can prevent antibiotic resistance.’ (P18)*

Other participants had some suggestions about how physicians, pharmacists and policymakers should prevent ABR. A few proposed that physicians should only prescribe antibiotics when they are needed, and should educate patients about ways to prevent ABR:

*‘In my view, doctors should prescribe antibiotics when patients really need them. Besides, they should explain what we should do to prevent [antibiotic] resistance.’ (P12)*

*‘In my opinion, doctors should give advice on how we can prevent antibiotic resistance. Most importantly, they should only prescribe antibiotics when patients need them.’ (P14)*

Two participants pointed out that policymakers should take steps to enforce the law on prescribing and dispensing antibiotics:

*‘I suppose the government should enforce the law so that antibiotics won’t be freely available in [community] pharmacies.’ (P17)*

*‘The government should do something so that doctors won’t simply prescribe antibiotics and [community] pharmacists stop selling the medicine without a prescription.’ (P20)*

Only one participant stated that pharmacists should educate patients on how to use antibiotics responsibly to prevent ABR:

*‘Pharmacists should provide an adequate explanation of how to take antibiotics appropriately and how to avoid [antibiotic] resistance.’ (P19)*

### Theme 4: perceptions of taking responsibility for combating ABR

When asked about the stakeholders who should take responsibility for combating ABR, the majority felt that physicians play a vital role in combating this major public health problem, as they are medical experts and have the authority to prescribe antibiotics:

*‘Apart from being trained in medical science, doctors are also qualified to prescribe antibiotics. Hence, they should take responsibility for combating antibiotic resistance.’ (P5)*

*‘In my view, doctors play a key role in combating antibiotic resistance because they are experts in the field.’ (P8)*

Some participants indicated that pharmacists should also take responsibility for combating ABR, because they are members of the healthcare team and dispense medicines:

*‘Pharmacists are part of the medical team. Their job is to prepare all sorts of medicines including antibiotics. So they should take responsibility for combating antibiotic resistance.’ (P3)*

*‘As pharmacists prepare medicines for patients, there is no doubt in my mind that they should take responsibility for combating antibiotic resistance.’ (P9)*

Only a few participants perceived themselves as having responsibility for preventing ABR, because they believed that they were at risk of contracting antibiotic-resistant infections:

*‘As a consumer, I get the feeling that I could be affected by antibiotic resistance. Therefore, I should take action to prevent this problem.’ (P15)*

*‘I think all patients, including myself, have responsibility for combating [antibiotic] resistance because all of us are at risk of getting an antibiotic-resistant infection.’ (P18)*

Two participants emphasised that policymakers play a key role in combating ABR, as they develop policies and enforce the law:

*‘First and foremost, the government develops policies and has the power to enforce the law. Consequently, it plays a major role in combating antibiotic resistance.’ (P17)*

*‘The government is the one who develops policies and therefore it has to take the lead in combating antibiotic resistance.’ (P20)*

## Discussion

This qualitative study yields important findings regarding community residents’ knowledge, attitudes and perceptions in relation to antibiotics and ABR. It also identifies areas of focus that need to be addressed when developing an educational intervention to increase their knowledge and change their attitudes and perceptions.

The study revealed that only a few participants were aware that antibiotics are medicines used to treat bacterial infections. The majority asserted that antibiotics are effective for treating viral infections, while others understood them as medicines that reduce fevers and inflammation, relieve pain, or prevent any illnesses from becoming severe. This aligns with the findings of other Malaysian studies, whereby the majority of the public surveyed in the States of Selangor (68%) [56] and Penang (67%) [57] stated that antibiotics are effective against viral infections. Similarly, a large number (64–71%) of the public surveyed in Indonesia [58], Namibia [59], Nigeria [60], Pakistan [61], Saudi Arabia [62], South Korea [63], the United States [64] and across many countries [32] also indicated that antibiotics are medicines that kill viruses.

This could be attributed to several factors. First, the study participants might not be adequately informed by healthcare professionals about what antibiotics should be used for and when they should be used [65]. Second, physicians and pharmacists often use the term ‘germs’ rather than ‘bacteria’ or ‘viruses’ when educating the public about infections [57, 65, 66]. Finally, frequent antibiotic prescribing for upper respiratory tract infections (URTIs) could contribute to public misconceptions that antibiotics are the first-line therapy for such conditions and can rapidly improve their symptoms [57, 67, 68]. As a result, the public is inclined to expect or request antibiotics from physicians when they have symptoms of URTIs [67–69]. In this study, a few participants indicated that they would make their expectations explicit and request antibiotics from their physician when they had viral infections. Conversely, some noted that they would not express these expectations openly nor request antibiotics, as they would rather maintain a good relationship with their physician. This is similar to the findings of a UK study conducted by Butler et al. [70], whereby one-third of the public expected antibiotics from a physician when they had colds or sore throats, but only one participant made this expectation explicit. Others viewed the physician–patient relationship as important, and were thus reluctant to request antibiotics from their physician. Lam et al. [71] also reported that 22% of the public surveyed in Hong Kong expected their physician to prescribe antibiotics for viral infections. However, they did not make their expectations explicit. Healthcare professionals play a key role in educating patients about the appropriate use of antibiotics. This could be done during the clinical consultation by providing explanations as to why antibiotics are not needed (e.g. viral infections) [72]. In fact, at the clinical consultation level, discussing the benefits and harms of antibiotics in a balanced way is a useful strategy to enhance patients’ understanding of antibiotics. The discussion could include the indication of the antibiotics and the potential harms associated with ABR on the patient and the community [21, 73]. Therefore, the key role of healthcare professionals is central in the rational use of antibiotics.

This study revealed that most participants were unaware of the common adverse effects of antibiotics, while other Malaysian studies showed that slightly more than half of the public surveyed in Penang (54%) [57] and the Federal Territory of Putrajaya (52%) [74] did not know that antibiotics have adverse effects. Similarly, Barber et al. [75] found that 55% of the public surveyed in the Philippines were unaware of the adverse effects of antibiotics. This might indicate that pharmacists are not educating patients about the common adverse effects of antibiotics and possible allergic reactions, how these can be minimised and what action they should take when these adverse effects occur [76, 77]. Inaccurate or inadequate information could influence public attitudes towards antibiotics. Some patients may be reluctant to take antibiotics because they fear adverse effects [78–80].

Most of the study participants acquired antibiotics from their physician. This could be due to the fact that in Malaysia, most medicines are available free of charge in public hospitals and primary-care clinics [81, 82]. Private practice physicians also have the legal right to prescribe and dispense medicines. Consequently, most patients obtain medicines from hospitals or primary-care clinics rather than community pharmacies [83, 84]. Some participants reported that they acquired antibiotics from a community pharmacy before consulting a physician, as they wanted to recover quickly from viral infections. This aligns with the findings of a public survey conducted in Kenya, whereby 22% of participants with acute respiratory tract infections acquired antibiotics from a community pharmacy before seeking the advice of a physician [85]. In this study, a few participants took antibiotics given to them by their family or friends, as they believed that antibiotics are effective for treating colds and sore throats. This practice has also been reported in Indonesia [25], Nigeria [86], Palestine [87] and other LMICs [72]. Similar to studies conducted in Indonesia [25], Jordan [88–90], the UK [28] and other LMICs [72], a few participants in the present study reported that they took leftover antibiotics prescribed for a previous illness when similar symptoms arose, because they wanted to save the time it would take to visit a primary-care clinic. Self-medication with antibiotics is a cause for concern because in Malaysia, antibiotics are classified as prescription-only medicines—therefore, dispensing them without a prescription is prohibited [91]. In addition, this practice not only contributes to the emergence and spread of ABR, but can also mask the diagnosis of infectious diseases and the development of chronic diseases. It may also result in adverse drug reactions and high treatment costs, since non-prescribed antibiotic use is usually associated with inappropriate medicine selection and suboptimal dosing or treatment duration [92–97]. Thus, the public must gain a better understanding of why antibiotics should only be taken when they have been prescribed to a specific individual for a particular illness [32]; pharmacists should recommend OTC medicines rather than antibiotics for minor illnesses such as colds [10, 98]; and policymakers should take steps to strengthen implementation of regulations on dispensing antibiotics [18, 98, 99].

The WHO [32] suggests that patients should adhere to antibiotic regimens and take the full prescribed course. This study showed that more than half of the participants took antibiotics according to the instructions on the label. However, they intentionally stopped taking the medicine when their symptoms improved, because they believed that they had recovered from the infection when they felt better. This is consistent with the findings of other Malaysian studies, whereby the majority of the public surveyed in the State of Pahang (69%) [8] and the Federal Territory of Kuala Lumpur (55%) [100] did not complete their prescribed course of antibiotics. Similarly, more than half (57–71%) of the public surveyed in India [101–103] and Saudi Arabia [104, 105] discontinued taking antibiotics when they felt better. On the contrary, less than one-third (30%) of the public surveyed in Hong Kong [106], Oman [107] and Qatar [108] stopped taking antibiotics when their symptoms improved. This suggests that pharmacists should educate patients about the importance of taking a full prescribed course of antibiotics [99]. Providing clear instructions and adequate explanations will positively influence their adherence to antibiotic therapy [109].

This study revealed that some participants occasionally missed a few doses of antibiotics because of their hectic lifestyle. Forgetting to take antibiotics indicates that participants had forgotten that they were feeling unwell or believed their condition was not severe, meaning that antibiotics were not needed or their condition had improved to the point that it was no longer necessary to take antibiotics [28, 110].

Most of the study participants disposed of leftover antibiotics in a dustbin; none returned them to pharmacists or physicians. This aligns with the findings of other Malaysian studies, whereby the majority of the public surveyed in Kuala Lumpur (79%) [100] and Pahang (64%) [8] disposed of leftover antibiotics in a rubbish bin. Tong et al. [111] also reported that the most common method for disposing of leftover medicines was placing them in a dustbin (43%). Disposing of leftover antibiotics in this manner can cause harm to children, pets and individuals who intentionally search household trash seeking medicines. Moreover, these antibiotics could enter the environment through landfill leachates and eventually contaminate water systems, resulting in ABR [112–115]. Hence, pharmacists should encourage patients to return leftover antibiotics to any public hospital or primary-care clinic when their physician advises them to stop taking their prescribed course [116]. Further, they should educate patients regarding the consequences of improper antibiotic disposal.

The study revealed that most participants had heard of the term ‘ABR’, but only a few correctly understood the term as a condition where bacteria become resistant to antibiotics. The majority understood the term as the body becoming used to antibiotics, whereas others understood it as antibiotics losing their potency before killing bacteria, the body becoming incompatible with antibiotics, the illness becoming too strong for antibiotics to manage or a hereditary illness. This is consistent with the findings of qualitative studies carried out in European countries [17], India [18] and the UK [26], and the findings of public surveys conducted in Kuwait [117], Sweden [118] and across many countries [32], whereby the majority of participants (56–87%) asserted that ABR occurs when the body becomes used to antibiotics. This common misconception could raise public concerns about personal overuse of antibiotics and deter them from completing a prescribed course [19, 28].

Although most of the study participants were aware of a correlation between antibiotic use and ABR, they remained uncertain about the causes of this problem. Some attributed ABR to external factors, such as inappropriate prescribing or dispensing of antibiotics without a prescription, rather than internal factors, such as non-adherence to antibiotic therapy. In addition, they believed that they were not at risk of contracting antibiotic-resistant infections, despite the fact that antibiotic-resistant bacteria can spread readily from one person to another and affect anyone [119, 120]. As a consequence, they neither perceived themselves as being responsible for preventing ABR, nor indicated that they play an important role in avoiding the problem. This is similar to the findings from other qualitative studies conducted in the UK [26, 27], whereby most of the public perceived the causes of ABR as due to external rather than internal factors. As a result, they believed that others should take action to prevent ABR. This is a cause for concern, as all stakeholders, including the public, should take responsibility for combating this problem [3, 5, 119, 121].

The study showed that most participants were unaware of the consequences of ABR, and not concerned about this issue. Similar findings have been reported in the UK [26, 27], whereby the majority of the public was unclear about the impact of ABR on individuals and the community. Conversely, a public survey conducted in the UK [122] indicated that 81% of participants were concerned about ABR after participating in public education campaigns on antibiotics.

### Study strengths and limitations

The purposive and maximum variation sampling was employed in this study. In fact, this sampling strategy can achieve representativeness and capture the heterogeneity in the population. Thus, the study managed to capture a wide range of community residents’ views, knowledge, attitudes and perceptions regarding antibiotics and ABR. Hence, it provided a greater insight into the study phenomena [49]. In addition, a rigorous approach was used to ensure the data validity and reliability by verifying the transcripts and study findings from the participants, and by involving an independent researcher in the analysis [123–125]. The small sample size might be seen as a limitation when judged by quantitative standards. However, the adequacy of sample in qualitative research is determined by data saturation—which is the gold standard method that determines the adequacy of the sample size in qualitative research [126]. In addition, our sample size is comparable to other studies, particularly the studies that used the same methodology (i.e. qualitative study) and the same topic [20, 21, 24–26, 30]. As with most interview studies, it is possible that the study participants gave socially desirable responses or were unwilling to share their experiences, attitudes and perceptions openly [28]. To minimise this, the authors reassured the participants that no consequences would arise from anything they revealed during the interviews [127]. Moreover, as the majority had a misconception about the term antibiotics, they could have confused antibiotics with other medicines when talking about their previous experiences with antibiotics. However, we were aware of this issue and tried to minimise it by in-depth discussions to ensure that the participants were discussing their experiences with antibiotics. Additionally, the interviews were conducted in a professional manner—no one else was present and participant confidentiality was maintained. As the study was conducted in Penang, the findings may not be generalised throughout Malaysia. However, since the study participants were from different age groups, ethnic, educational and socioeconomic backgrounds, it is likely that diverse communities in other parts of Malaysia would have a similar lack of knowledge about antibiotics and ABR. Overall, we believe that the findings are robust and might be transferable to similar populations or settings in Malaysia. However, more studies to replicate the results in different settings, populations or contexts are needed as other aspects and issues surrounding the use of antibiotics might be uncovered.

## Conclusions

The findings indicate that the majority of community residents lacked knowledge about antibiotics and ABR, had negative attitudes towards antibiotics and had negative perceptions of ABR. Therefore, there is an urgent need to develop and implement educational interventions to address key gaps in their knowledge, correct common misconceptions and shift their attitudes and perceptions. The areas of focus that need to be addressed include the appropriate use of antibiotics and their adverse effects; the importance of adhering to antibiotic therapy; and the definition, causes, consequences and prevention of ABR.

## Supplementary information

**Additional file 1.** Interview guide

## Data Availability

The datasets generated or analysed during the study are available from the corresponding author on reasonable request.

## Abbreviations

ABR: Antibiotic Resistance
LMICs: Low-and Middle-Income Countries
MYR: Malaysian Ringgit
OTC: Over the Counter
UK: United Kingdom
URTIs: Upper Respiratory Tract Infections
USM: Universiti Sains Malaysia
WHO: World Health Organization
