# Supplementary material for: Low-income community knowledge, attitudes and perceptions regarding antibiotics and antibiotic resistance in Jelutong District, Penang, Malaysia: a qualitative study
Source: BMC Public Health. 2019 Oct 15;19:1292. doi: 10.1186/s12889-019-7718-9 (PMC6794866; doi:10.1186/s12889-019-7718-9)
Supplement: Supplementary file 1 — Additional file 1. Interview guide [file 12889_2019_7718_MOESM1_ESM.docx]

**Additional file 1** Interview guide

**Part I. Knowledge of and attitudes towards antibiotics**

1. What do you understand by the term ‘antibiotics’?

Prompting questions:

- Are you currently taking, or have you previously taken, antibiotics?
- What are antibiotics?

2. When are antibiotics used?

Prompting questions:

- Why do you take antibiotics?
- Which conditions can be treated with antibiotics?

3. How do you acquire antibiotics?

Prompting questions:

- Where do you get antibiotics?
- Do you share antibiotics with anyone else? Why?
- Do you keep a supply of antibiotics at home? Why?
- Do you take leftover antibiotics prescribed for your previous illness? Why?

Probing question:

- What would you do if your doctor did not prescribe antibiotics for you?

4. How do you take antibiotics?

Prompting questions:

- Do you get advice from your doctor or pharmacist on how to take antibiotics? Please share your experience.
- Do you take antibiotics according to the instructions on the label? Why?
- What would you do if you missed a dose of antibiotics?
- Do you finish the prescribed course of antibiotics? Why?
- When do you stop taking antibiotics? Why?
- What do you do with leftover antibiotics?
- How do you dispose of leftover antibiotics?

5. Do you know if antibiotics have any side effects? If so, what are they?

Prompting questions:

- Have you experienced any side effects while taking antibiotics? Please share your experience.
- What action would you take when these side effects occur?

**Part II. Knowledge and perceptions of antibiotic resistance**

1. What do you understand by the term ‘antibiotic resistance’?

Prompting questions:

- Have you heard of the term ‘antibiotic resistance’?
- How did you hear about it?
- Has antibiotic resistance ever been a topic of conversation with your family or friends?

Probing question:

- Some people believe that human bodies can become resistant to antibiotics. To what extent do you agree with this statement?

2. What are the causes of antibiotic resistance?

Probing question:

- Do you think there is an association between antibiotic use and antibiotic resistance? Please share your thoughts.

3. What are the consequences of antibiotic resistance?

Probing question:

- Do you think infections are more difficult to treat because of antibiotic resistance? Please share your thoughts.

4. How can antibiotic resistance be prevented?

Prompting question:

- How do you prevent antibiotic resistance?

Probing question:

- Do you think antibiotic resistance could affect you or your family? Please share your throughts.

5. Who should take responsibility for combatting antibiotic resistance? Why?

Probing question:

- Do you think you should take responsibility for combatting antibiotic resistance? Please share your thoughts.

Are there any additional opinions you would like to express regarding antibiotics and antibiotic resistance?
